# Prevalence, probability, and characteristics of malaria and filariasis co-infections: A systematic review and meta-analysis

**DOI:** 10.1371/journal.pntd.0010857

**Published:** 2022-10-21

**Authors:** Polrat Wilairatana, Kwuntida Uthaisar Kotepui, Wanida Mala, Kinley Wangdi, Manas Kotepui

**Affiliations:** 1 Department of Clinical Tropical Medicine, Faculty of Tropical Medicine, Mahidol University, Bangkok, Thailand; 2 Medical Technology, School of Allied Health Sciences, Walailak University, Tha Sala, Nakhon Si Thammarat, Thailand; 3 Department of Global Health, National Centre for Epidemiology and Population Health, College of Health & Medicine, Australian National University, Canberra, Acton, Australian Central Territory, Australia; Minia University, EGYPT

## Abstract

**Background:**

Malaria and filariasis are significant vector-borne diseases that are co-endemic in the same human populations. This study aims to collate the evidence, probability, and characteristics of malaria and filariasis co-infections in participants among studies reporting the co-occurrence of both diseases.

**Methods:**

We searched for potentially relevant articles reporting the co-occurrence of malaria and filariasis in five electronic databases (Embase, PubMed, Scopus, Medline, and CENTRAL) from inception to May 22, 2022. We estimated the pooled prevalence and probability of malaria and filariasis co-infections among study participants using random-effects meta-analyses and synthesized the characteristics of patients with co-infections narratively.

**Results:**

We identified 951 articles, 24 of which (96,838 participants) met eligibility criteria and were included in the systematic review. Results of the meta-analysis showed a pooled prevalence of malaria and filariasis co-infections among participants of 11%. The prevalence of co-infections was 2.3% in Africa, 0.2% in Asia, and 1.6% in South America. The pooled prevalences of malaria and *Wuchereria bancrofti*, malaria and *Loa loa*, malaria and *Mansonella perstans* co-infections were 0.7%, 1.2%, and 1.0%, respectively. The meta-analysis results showed that the co-infections between two parasites occurred by probability (*P* = 0.001). Patients with co-infections were at increased risk of having an enlarged spleen, a lower rate of severe anemia, lower parasite density, and more asymptomatic clinical status. Patients with co-infections had decreased levels of C-X-C motif chemokine 5, tumor necrosis factor–α, interleukin-4, c4 complement, and interleukin-10. In addition, patients with co-infections had a lower interleukin-10/tumor necrosis factor–α ratio and higher interleukin-10/interleukin-6 ratio.

**Conclusion:**

The present study showed that the prevalence of malaria and filariasis co-infections was low and varied between geographical areas in the selected articles. Co-infections tended to occur with a low probability. Further studies investigating the outcomes and characteristics of co-infections are needed.

## Author summary

Malaria is caused by the bite of *Anopheles* mosquitoes infected with the protozoan genus *Plasmodium* species. Filariasis is caused by the infections of filarial nematode diseases, including lymphatic filariasis, mansonellosis, and loiasis through the bite of *Anopheles* and other vectors. We searched and synthesized the literatures reporting the co-occurrence of malaria and filariasis. We found that the overall prevalence of malaria and filariasis co-infections among participants of 11%. The prevalence of co-infections was 2.3% in Africa, 0.2% in Asia, and 1.6% in South America. The overall prevalences of malaria and lymphatic filariasis, malaria and loiasis, malaria and mansonellosis co-infections were 0.7%, 1.2%, and 1.0%, respectively. The co-infections between two parasites occurred by probability. Patients with co-infections of both diseases were at increased risk of having an enlarged spleen, a lower rate of severe anemia, lower parasite density, and more asymptomatic clinical status. In addition, patients with co-infections showed alterations of cytokine and chemokine levels such as C-X-C motif chemokine 5, tumor necrosis factor–α, interleukin-4, c4 complement, interleukin-6, and interleukin-10.

## Introduction

Malaria is a disease caused by the protozoa genus *Plasmodium* species, including *P*. *falciparum*, *P*. *vivax*, *P*. *ovale curtisi*, *P*. *ovale wallikeri*, *P*. *malariae*, and *P*. *knowlesi* [1]. According to World Health Organization reports in 2022, there were an estimated 241 million malaria cases and 627,000 deaths in 2020 [2]. Filarial nematode diseases, including lymphatic filariasis (caused by *Wuchereria bancrofti*), onchocerciasis (caused by *Onchocerca volvulus*), and loiasis (caused by *Loa loa*) are the most common filarial infections in sub-Saharan Africa [3–5]. In addition to *Wuchereria bancrofti*, lymphatic filariasis can be caused by *Brugia malayi* and *Brugia timori* in a lesser proportion [6]. Approximately 51 million people in Africa, southeast Asia, the Pacific, the Caribbean, South America, and the Middle East were infected with lymphatic filariasis [7,8]; in addition, more than 29 million people are at risk of contracting loiasis in Central and West Africa [9]. Another filarial parasite, *Mansonella perstans*, is the cause of Mansonellosis, and most patients infected with *M*. *perstans* have been reported to be asymptomatic, unlike those with loiasis [10].

Malaria and filariasis are significant vector-borne diseases that are co-endemic in the same human populations. The common vectors are *Anopheles gambiae*, *An*. *arabiensis*, *An*. *merus*, *An*. *merus*, *An*. *funestus in Afroca; An*. *philippinensis* and *An*. *barbirostris* in India and Bangladesh; *An*. *leucosphyrus*, *An*. *barbirostris*, *An*. *donaldi*, *An*. *letifer*, *An*. *whartoni*, *An*. *macualtus*, *An*. *campestris* in Malaysia; *An*. *minimus* in the Philippines; *An*. *balabacensis* in Indonesia; *An*. *farauti* and *An*. *punctulatus* in Papua New Guinea; *An*. *koriensis* in the Solomon Islands; *An*. *darlingi* and *An*. *aquasalis* in South America; and *An*. *sinensis* in China and Korea [11]. Consequently, residents of regions such as Africa and Asia, where both malaria and filariasis are endemic, continue to be at risk of contracting and experiencing morbidity associated with both diseases. The World Health Organization is currently implementing integrated vector management targeted at these vectors to reduce pathogen transmission and hence reduce disease burden [12]. To date, there is no available evidence-based information on the prevalence of concurrent infections of the two diseases in human populations. It is more likely that the control of either malaria or filaria parasitemia would reduce the number of mosquitoes carrying the pathogens as well as the transmission of infectious diseases [13]. Because co-infection and interaction phenomena in human populations are complex, it is necessary to comprehend co-infection status and their characteristics. To obtain information on the occurrence, distribution, and prevalence of co-infections of the two diseases, and to collect baseline data on which efforts toward designation and implementation of an integrated control strategy may be based, the present study aimed to collate the evidence and characteristics of malaria and filariasis co-infections in participants among studies reporting the co-occurrence of both diseases.

## Methods

### Protocol and registration

The systematic review protocol was registered at PROSPERO (registration No. CRD42022334494). The results of the systematic review and meta-analysis followed the Preferred Reporting Items for Systematic Reviews and Meta-Analyses (PRISMA) 2020 statement [14].

### Research questions

The systematic review question followed the PICO (Participant, Intervention, Comparator, Outcome) question. P included participants who were enrolled in the studies for testing malaria and filariasis parasites by any method, I was not applied, C was not applied, and O was the prevalence of co-infections.

### Search strategy

We searched five electronic databases (Embase, PubMed, Scopus, Medline, and CENTRAL) for peer-reviewed articles published between January 1, 1898, and May 22, 2022, in the English language. An additional search was performed in Google Scholar. The search strategy used the following key terms and their appropriate synonyms: (1) malaria, AND (2) filariasis, AND (3) (coinfect* OR coinfect-infect* OR concurent* OR mix* OR co-occur* OR coincident OR coincidental OR coinciding OR cooccur* OR simultaneous). For searching in PubMed, we used Medical Subject Heading terms to help search with key terms to retrieve potentially relevant studies. S1 Table shows the complete search strategy and filter for each database. Synonyms of each key term were identified from the Medical Subject Heading. Relevant studies were also searched in the reference lists of the included studies and Google Scholar.

### Eligibility criteria

We included primary observational studies (prospective or retrospective) and cross-sectional studies to examine the pooled prevalence of malaria and filariasis co-infections among enrolled human participants. In addition, we included cohort and case-control studies to identify the difference in characteristics between co-infections and malaria/filariasis monoinfection. Malaria is diagnosed by microscopy, rapid diagnostic test (RDT), or molecular methods. Filariasis is diagnosed by direct microscopy, leukoconcentration, circulating filarial antigen (RDT), or molecular methods. A patient was considered to have concomitant infections of malaria and filariasis if positive for both malaria and filaria parasites by the above tests. We excluded studies without full-text (i.e., full texts were unavailable for evaluation by the authors as a limitation on the access to the full texts), review articles (without original data of co-infections); case reports; mosquito studies; studies reporting malaria intervention measures, vector co-infections, transmission dynamics, or the development of techniques for malaria/filariasis identifications; studies from which we were unable to extract the data of co-infections; studies with spatial statistics demonstrating co-infections; in vivo or in vivo studies; studies conducted by the same authors with overlapping participants; and conference abstracts.

### Study selection and data extraction

Two review authors (M.K., K.U.K.) independently performed the study selections. All titles and abstracts, followed by the full texts of potentially relevant studies, were screened, and relevant studies were included for full-text screening against eligibility criteria. Discrepancies between the two review authors during the study selection process were resolved by discussion to reach a consensus. Two reviewer authors (M.K., K.U.K.) extracted the following data into a Microsoft Excel spreadsheet (Microsoft Corporation, Redmond, WA, USA): names of the first authors, publication year, study sites, time for conducting the study, study design, number and characteristics of participants, number and characteristics of patients with co-infections, malaria and filariasis parasites, characteristics of co-infections, and diagnostic method for malaria and filariasis parasites.

### Quality assessment

Two review authors (M.K., K.U.K.) assessed the quality of all included studies using the critical appraisal tools of the Joanna Briggs Institute (JBI) [15]. The JBI tools for prevalence and analytical cross-sectional studies assess the quality of observational and cross-sectional studies. We used the JBI tools for case-control and cohort studies to determine the quality of those study designs. The quality of the included studies was rated using the percentile as described previously [16,17].

### Data synthesis

We estimated the pooled prevalence and probability of malaria and filariasis co-infections among participants included in the studies using random-effects meta-analyses by DerSimonian and Laird [18]. The remaining studies, including case-control, cohort studies, and observational studies that could not be included for quantitative synthesis,. were synthesized narratively. We used forest plots to display (1) the prevalence estimates and odds ratio (OR) of each study, (2) the pooled prevalence estimate and pooled OR from all studies, (3) estimated statistical heterogeneity (*I*^2^), (4) the weight of each study, and (5) the number of cases for meta-analysis of the pooled prevalence. To identify the possible source(s) of heterogeneity of the pooled prevalence, we conducted subgroup analyses using study area, study design, participants, febrile conditions, age groups, diagnostic methods for malaria, and diagnostic methods for filariasis. Publication bias was assessed by visualizing the funnel plot asymmetry, Egger’s test, and the contour-enhanced funnel plot. STATA 14.2 was used for all statistical analyses (StataCorp, College Station, TX, USA).

## Results

### Search results

**Fig 1:**
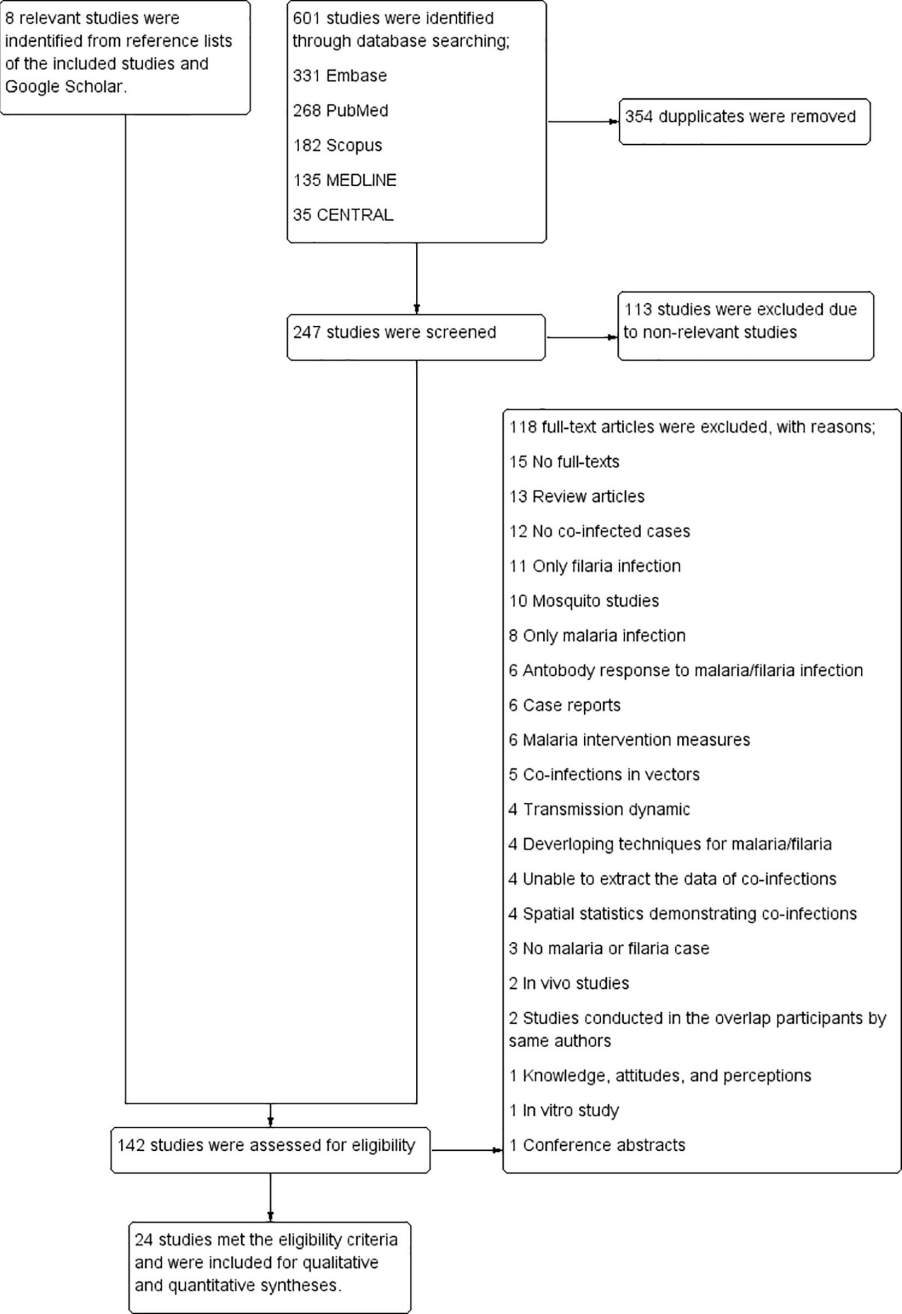
Study flow diagram.

A total of 951 articles were identified through database searching: 331 from Embase, 268 from PubMed, 182 from Scopus, 135 from Medline, and 35 from CENTRAL. After 352 duplicates were removed, 599 articles were screened for titles and abstracts. Then, the remaining 465 articles were excluded due to nonrelevant studies. The remaining 136 articles from five databases and eight relevant articles identified from Google Scholar were assessed for eligibility. A total of 118 full-text articles were excluded, with reasons: 15 without full texts, 13 review articles, 12 with no co-infected cases, 11 with only filariasis infections, 10 mosquito studies, 8 with only malaria infection, 6 antibody responses to malaria/filariasis infection, 6 case reports, 6 malaria intervention measures, 5 co-infections in vectors, 4 transmission dynamics, 4 developing techniques for malaria/filariasis identifications, 4 from which we were unable to extract the data of co-infections, 4 spatial statistics demonstrating co-infections, 3 with no malaria or filariasis case, 2 in vivo studies, 2 studies conducted by the same authors with overlapping participants, 1 knowledge/attitudes/perceptions, 1 in vitro study, and 1 conference abstract. The 24 articles that met the eligibility criteria were included in the systematic review (Fig 1).

### Characteristics of the included studies

**Fig 2:**
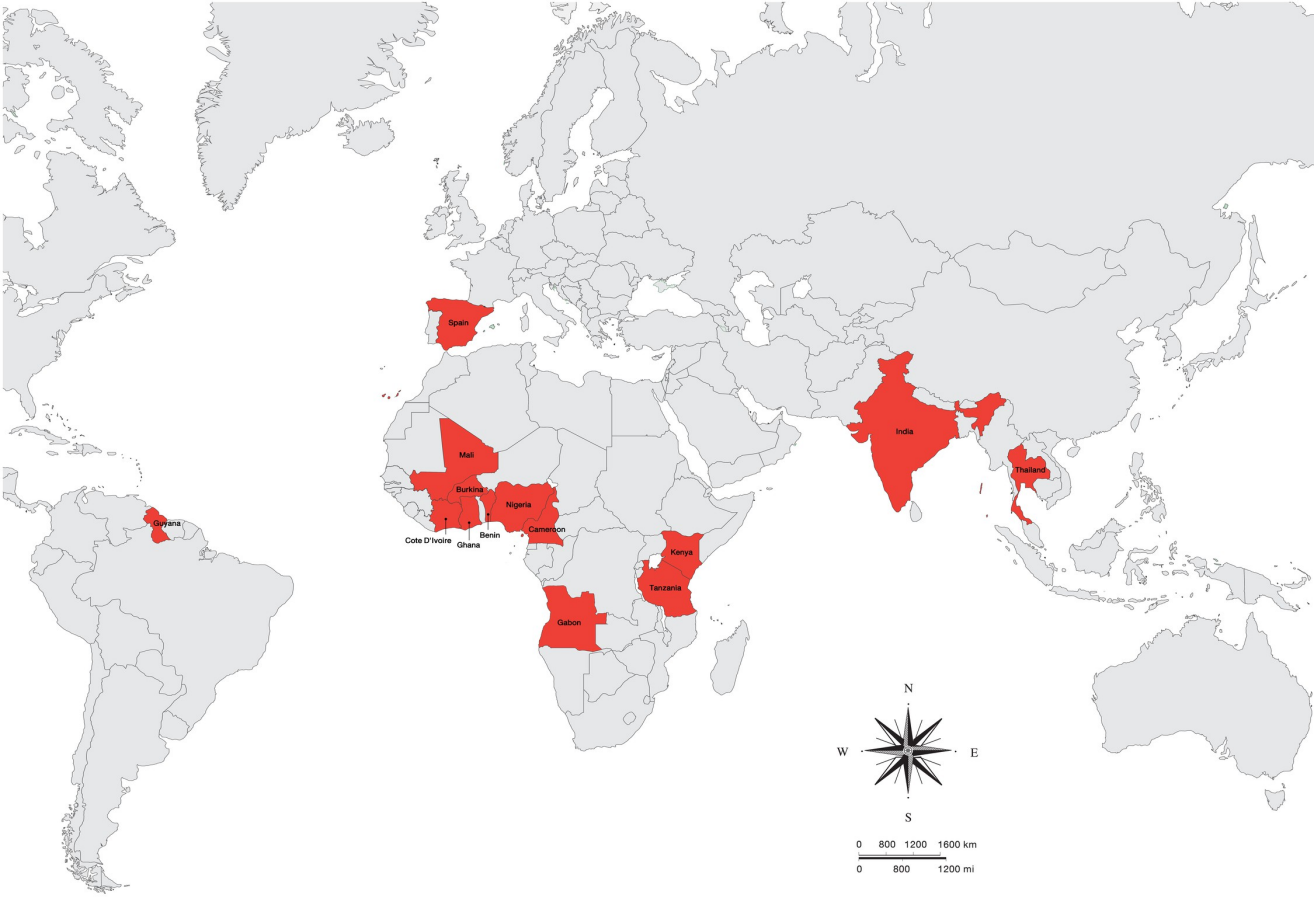
Geographic distribution of malaria and filariasis co-infections. The crimson color indicated countries where co-infections were reported.

Table 1 shows the characteristics of the included studies. The included studies were published between 1990 and 2022 and included cross-sectional studies (15, 62.6%), prospective observational studies (4, 16.7%), retrospective observational studies (2, 8.33%), cohort studies (2, 8.33%), and a (1, 4.17%) case-control study. The included studies were conducted in Africa (17, 70.8%), Asia (5, 20.8%), Europe (1, 4.17%), and South America (1, 4.17%; Fig 2). The included studies enrolled participants in communities (15, 62.5%), participants in hospitals/clinics (4, 16.7%), malaria-positive patients (2, 8.33%), malaria- and filariasis-positive patients (1, 4.17%), patients with HIV and filariasis (1, 4.17%), and imported malaria from sub-Saharan Africa (1, 4.17%). Most of the included studies enrolled participants of all ages (17, 70.8%). Most of the included studies used the microscopic method to identify malaria (14, 58.3%) and filariasis (9, 37.5%). S2 Table shows the details of the included studies.

**Table 1:** Characteristics of 24 studies included in the study.

Characteristics	N.	%
**Study designs**		
Cross-sectional studies	15	62.6
Prospective observational studies	4	16.7
Retrospective observational studies	2	8.33
Cohort studies	2	8.33
Case-control study	1	4.17
**Study areas**		
Africa	17	70.8
Asia	5	20.8
South America	1	4.17
Europe (imported malaria)	1	4.17
**Participants**		
Participants in communities	15	62.5
Participants in hospitals/clinics	4	16.7
Malaria-positive patients	2	8.33
Malaria and filariasis-positive patients	1	4.17
HIV and filariasis patients	1	4.17
Imported malaria from sub-Saharan Africa	1	4.17
**Age groups**		
All age groups	17	70.8
Children	4	16.7
Adults	3	12.5
**Methods for identifying malaria**		
Microscopy	14	58.3
Molecular method	3	12.5
Microscopy/RDT	2	8.33
RDT/molecular method	2	8.33
Microscopy/PfHRP2 ELISA	1	4.17
Microscopy/molecular method	1	4.17
Microscopy/RDT/molecular method	1	4.17
**Methods for identifying filariasis**		
Microscopy	9	37.5
Molecular method	4	16.7
Microscopy/RDT	4	16.7
RDT	3	12.5
Direct microscopy/Leukoconcentration technique	2	8.33
Microscopy for *M*. *perstans*, ELISA for *W*. *bancrofti*	1	4.17
Not specified	1	4.17

**Abbreviation:** ELISA- enzyme-linked immunosorbent assay; HIV- Human immunodeficiency virus; RDT- rapid diagnostic test

### Quality of the included studies

One cohort study was of high quality [19], and another was of moderate quality [20] because of the unclear follow-up time, lack of description of the loss to follow-up, and lack of strategies to address incomplete follow-up. One case-control study [21] was of moderate quality because it did not identify or mention the confounding factors and it lacked information on the exposure period of interest. Ten cross-sectional studies were of high quality; meanwhile, others were of moderate quality. All prospective and retrospective observational studies were of high quality. All studies were included in the systematic review (S3 Table).

### Prevalence of malaria and filariasis co-infections among selected studies

**Fig 3:**
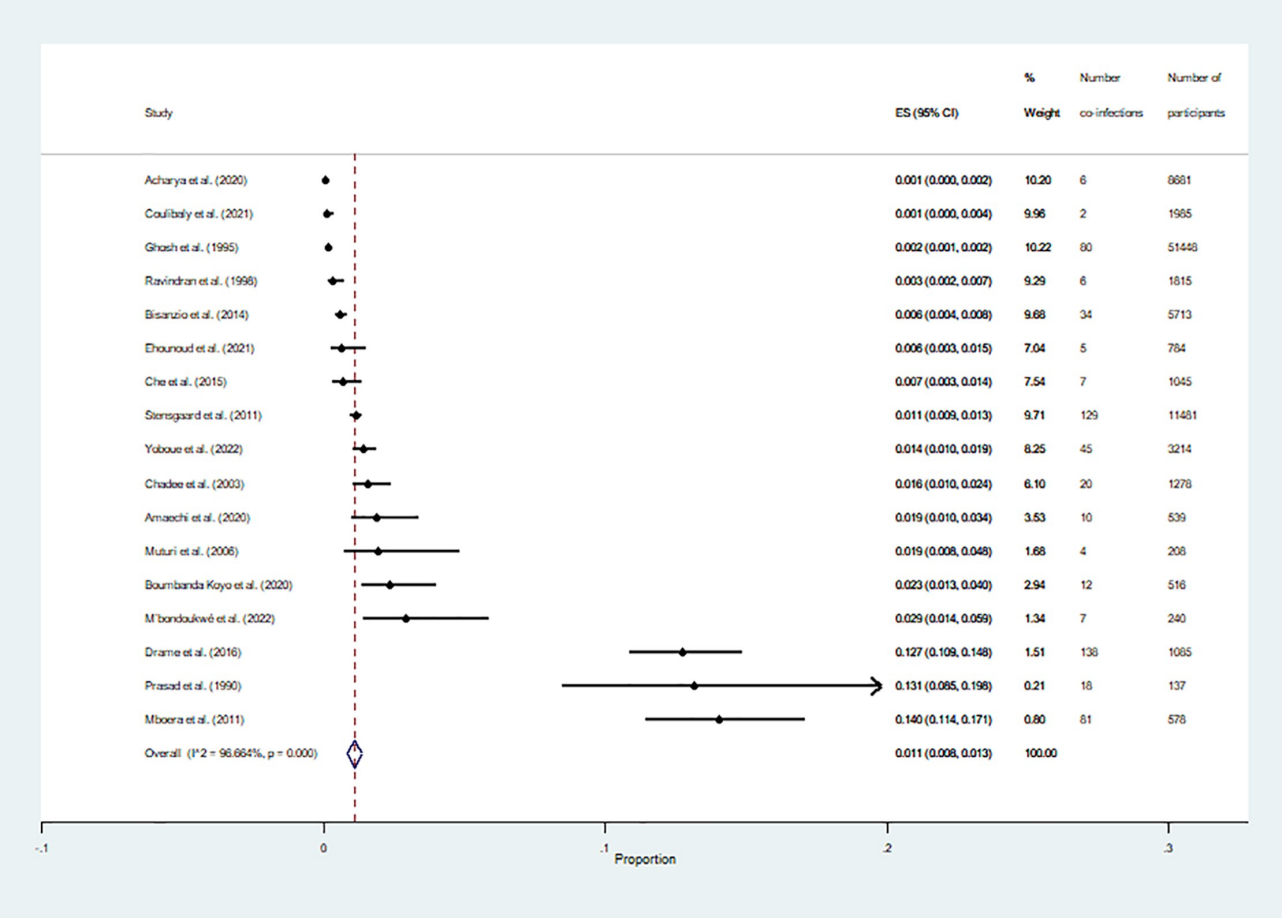
Pooled prevalence of malaria and filariasis co-infection among selected studies. **Abbreviations**: ES- prevalence estimate; CI- confidence interval.

We estimated the pooled prevalence of malaria and filariasis co-infections among participants using the data available from 17 studies [22–38]. Results of the meta-analysis showed that the pooled prevalence of malaria and filariasis co-infections among participants was 11% (95% confidence interval (CI): 8%–13%, *I*^2^, 96.7%, 17 studies; Fig 3). The highest prevalence was demonstrated by the study conducted in Tanzania (14%, 95% CI: 11.4%–17.1%) [33], whereas the lowest prevalence was demonstrated by studies conducted in India (0.1%, 95% CI: 0%–0.2%) [22] and in Burkina Faso (0.1%, 95% CI: 0%–0.4%) [27].

Table 2 shows the results of the subgroup analyses. Subgroup analysis of continents demonstrated that the prevalence of co-infections was 2.3% in Africa (95% CI: 1.6–2.9, *I*^2^: 96.8%, 12 studies), 0.2% in Asia (95% CI: 0–0.3, *I*^2^: 89.7%, 4 studies), and 1.6% in South America (95% CI: 1.0–2.4, 1 study). Subgroup analysis of the study designs demonstrated that the prevalence of co-infections among participants enrolled in cross-sectional, prospective observational, and retrospective observational studies was 1.5% (95% CI: 1.1–2.0, *I*^2^: 97%, 14 studies), 0.1% (95% CI: 0–0.1, *I*^2^: 99.8%, 2 studies), and 2.3% (95% CI: 1.3–1.0, 1 study), respectively. Subgroup analysis of participants demonstrated that the prevalence of co-infections among participants in communities and participants in hospitals/clinics was 1.6% (95% CI: 1.2–2.1, *I*^2^: 97.2%, 13 studies) and 1.0% (95% CI: 0.1–1.9, *I*^2^: 91.1%, 4 studies), respectively. Subgroup analysis of febrile conditions demonstrated that the prevalence of co-infections among febrile, both febrile and afebrile, and not specified for febrile conditions was 0.2% (95% CI: 0–0.3, *I*^2^: 91.6%, 3 studies), 1.0% (95% CI: 0.3–1.6, *I*^2^: 65.9%, 3 studies), and 2.6% (95% CI: 1.8–3.3, *I*^2^: 97.2%, 11 studies), respectively. Subgroup analysis of age groups demonstrated that the prevalence of co-infections among studies enrolling participants of all age groups and only children was 0.8% (95% CI: 0.5–1.0, *I*^2^: 95.4%, 14 studies) and 5.5% (95% CI: 1.0–10.0, *I*^2^: 97.6%, 3 studies), respectively. Subgroup analysis of diagnostic methods used for malaria demonstrated that the prevalence of co-infections among studies that used the microscopic method, molecular method, microscopy/RDT, RDT/molecular method, and microscopy/molecular method was 1.2% (95% CI: 0.7–1.7, *I*^2^: 96.6%, 9 studies), 5.1% (95% CI: 0–10.8, *I*^2^: 98.5%, 3 studies), 0.1% (95% CI: 0–0.1, *I*^2^: 99.3%, 2 studies), 0.8% (95% CI: 0.6–0.9, *I*^2^: 99.3%, 2 studies), and 2.9% (95% CI: 1.4–5.9, 1 study), respectively. Subgroup analysis of diagnostic methods for filariasis parasites demonstrated that the prevalence of co-infections among studies that used microscopic method, molecular method, direct microscopy/leukoconcentration technique, RDT, and microscopy/RDT was 0.3% (95% CI: 0.1–0.4, *I*^2^: 87.1%, 8 studies), 4.0% (95% CI: 1.5–6.5, *I*^2^: 97.8%, 4 studies), 2.9% (95% CI: 1.4–5.9, 1 study), 0.9% (95% CI: 0.7–1.0, *I*^2^: 99.3%, 2 studies), and 3.6% (95% CI: 2.5–4.6, *I*^2^: 99.3%, 2 studies), respectively.

**Table 2:** Subgroup analyses of the prevalence of malaria and filariasis co-infections among selected studies.

Subgroup	Prevalence estimate (%)	95% CI (%)	*I* ^2^	Number of studies
**Continents**				
Africa	2.3	1.6–2.9	96.8	12
Asia	0.2	0–0.3	89.7	4
South America	1.6	1.0–2.4	NA	1
**Study design**				
Cross-sectional study	1.5	1.1–2.0	97	14
Prospective observational studies	0.1	0–0.1	99.8	2
Retrospective observational studies	2.3	1.3–4.0	NA	1
**Participants**				
Participants in communities	1.6	1.2–2.1	97.2	13
Participants in hospitals/clinics	1.0	0.1–1.9	91.1	4
**Febrile conditions**				
Febrile	0.2	0–0.3	91.6	3
Febrile and afebrile	1.0	0.3–1.6	65.9	3
Not specified	2.6	1.8–3.3	97.2	11
**Age groups**				
All age groups	0.8	0.5–1.0	95.4	14
Children	5.5	1.0–10.0	97.6	3
Adults	NA	NA	NA	NA
**Diagnostic methods for malaria**				
Microscopy	1.2	0.7–1.7	96.6	9
Molecular method	5.1	0–10.8	98.5	3
Microscopy/RDT	0.1	0–0.1	99.3	2
RDT/molecular method	0.8	0.6–0.9	99.3	2
Microscopy/molecular method	2.9	1.4–5.9	NA	1
**Diagnostic methods for filariasis**				
Microscopy	0.3	0.1–0.4	87.1	8
Molecular method	4.0	1.5–6.5	97.8	4
Direct microscopy/Leukoconcentration technique	2.9	1.4–5.9	NA	1
RDT	0.9	0.7–1.0	99.3	2
Microscopy/RDT	3.6	2.5–4.6	99.3	2

**Abbreviation:** CI- confidence interval; NA- not assessed; RDT- rapid diagnostic test

### Prevalence of malaria and *Wuchereria bancrofti* co-infections among selected studies

**Fig 4:**
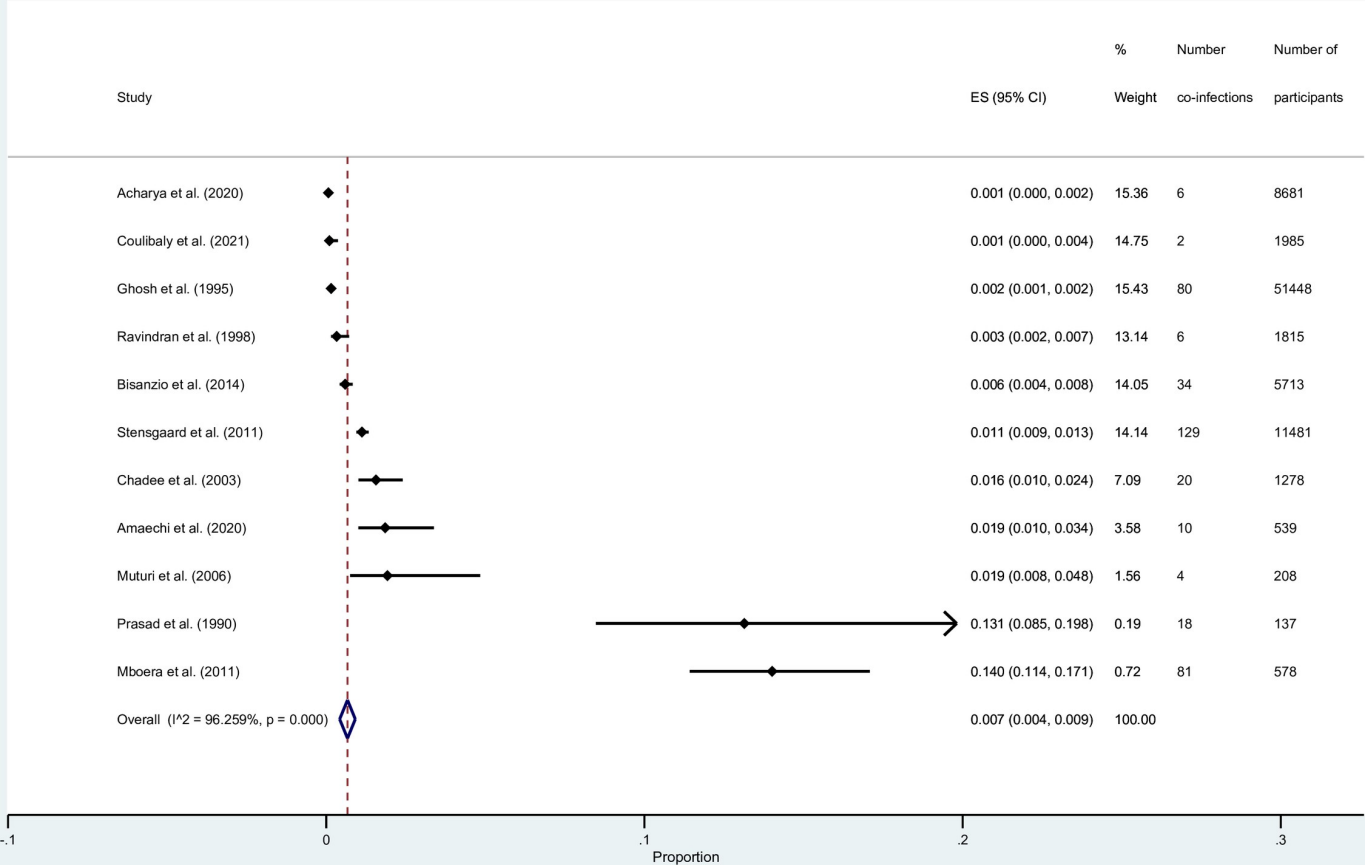
The prevalence of malaria and *Wuchereria bancrofti* co-infections among selected studies. **Abbreviations**: ES- prevalence estimate; CI:- confidence interval.

The pooled prevalence of malaria and *W*. *bancrofti* co-infections among participants was estimated using the data available from 11 studies [22,23,25,27,29,31,33–37]. The results of the meta-analysis showed that the pooled prevalence of malaria and *W*. *bancrofti* co-infections among participants was 0.7% (95% CI: 0.4%–0.9%, *I*^2^, 96.3%, 11 studies; Fig 4). The highest prevalence was demonstrated by the study conducted in Tanzania (14%, 95% CI: 11.4%–17.1%) [33], whereas the lowest prevalence was demonstrated by the study conducted in India (0.1%, 95% CI: 0%–0.2%) [22].

Results of subgroup analyses are shown in Table 3. Subgroup analysis of continents demonstrated that the prevalence of malaria and *W*. *bancrofti* co-infections among studies conducted in Africa, Asia, and South America were 1.7% (95% CI: 0.9–2.4, *I*^2^: 97%, 6 studies), 0.2% (95% CI: 0–0.3, *I*^2^: 89.7%, 4 studies), and 1.6% (95% CI: 1.0%–2.4%, 1 study), respectively. Subgroup analysis of study designs demonstrated that the prevalence of malaria and *W*. *bancrofti* co-infections among participants enrolled in cross-sectional and prospective observational studies was 1.0% (95% CI: 0.5–1.4, *I*^2^: 96.6%, 9 studies) and 0.1% (95% CI: 0–0.1, *I*^2^: 96.3%, 2 studies), respectively. Subgroup analysis of study designs demonstrated that the prevalence of malaria and *W*. *bancrofti* co-infections among studies that enrolled participants in communities and hospitals/clinics were 1.0% (95% *CI*: 0.5–1.4, *I*^2^: 96.6%, 9 studies) and 0.1% (95% CI: 0–0.1, *I*^2^: 96.3%, 2 studies), respectively. Subgroup analysis of febrile conditions demonstrated that the prevalence of malaria and *W*. *bancrofti* co-infections among studies that enrolled specified and nonspecified febrile conditions were not specified was 0.2% (95% CI: 0–0.3, *I*^2^: 91.6%, 3 studies) and 1.5% (95% CI: 0.9–2.1, *I*^2^: 96.3%, 8 studies), respectively. Subgroup analysis of age groups demonstrated that the prevalence of malaria and *W*. *bancrofti* co-infections among studies with participants of all age groups and those with children only was 0.3% (95% CI: 0.2–0.5, *I*^2^: 89.8%, 9 studies) and 1.2% (95% CI: 1.0–1.4, *I*^2^: 98.7%, 2 studies), respectively. Subgroup analysis of diagnostic methods for malaria demonstrated that the prevalence of malaria and *W*. *bancrofti* co-infections among studies that used the microscopic, microscopy/RDT, and RDT/molecular methods was 1.2% (95% CI: 0.7–1.7, *I*^2^: 96.6%, 9 studies), 0.1% (95% CI: 0–0.2, 1 study), and 0.6% (95% CI: 0.4–0.8, 1 study), respectively. Subgroup analysis of the diagnostic methods for filariasis parasites demonstrated that the prevalence of co-infections among studies that used the microscopic, RDT, and microscopy/RDT methods was 0.2% (95% CI: 0.1–0.4, *I*^2^: 88%, 7 studies), 0.9% (95% CI: 0.7–1.0, *I*^2^: 98%, 2 studies), and 3.6% (95% CI: 2.5–4.6, *I*^2^: 98%, 2 studies), respectively.

**Table 3:** Subgroup analyses of the prevalence of malaria and *Wuchereria bancrofti* co-infections among selected studies.

Subgroup	Prevalence estimate (%)	95% CI (%)	*I* ^2^	Number of studies
**Continents**				
Africa	1.7	0.9–2.4	97.0	6
Asia	0.2	0–0.3	89.7	4
South America	1.6	1.0–2.4	NA	1
**Study design**				
Cross-sectional studies	1.0	0.5–1.4	96.6	9
Prospective observational studies	0.1	0–0.1	96.3	2
**Participants**				
Participants in communities	1.0	0.5–1.4	96.6	9
Participants in hospitals/clinics	0.1	0–0.1	96.3	2
**Febrile conditions**				
Febrile	0.2	0–0.3	91.6	3
Not specified	1.5	0.9–2.1	96.3	8
**Age groups**				
All age groups	0.3	0.2–0.5	89.8	9
Children	1.2	1.0–1.4	98.7	2
**Diagnostic methods for malaria**				
Microscopy	1.2	0.7–1.7	96.6	9
Microscopy/RDT	0.1	0–0.2	NA	1
RDT/molecular method	0.6	0.4–0.8	NA	1
**Diagnostic methods for filariasis**				
Microscopy	0.2	0.1–0.4	88.0	7
RDT	0.9	0.7–1.0	98.0	2
Microscopy/RDT	3.6	2.5–4.6	98.0	2

**Abbreviation:** CI- confidence interval; NA- not assessed; RDT- rapid diagnostic test

### Prevalence of malaria and *L*. *loa* co-infections among selected studies

**Fig 5:**
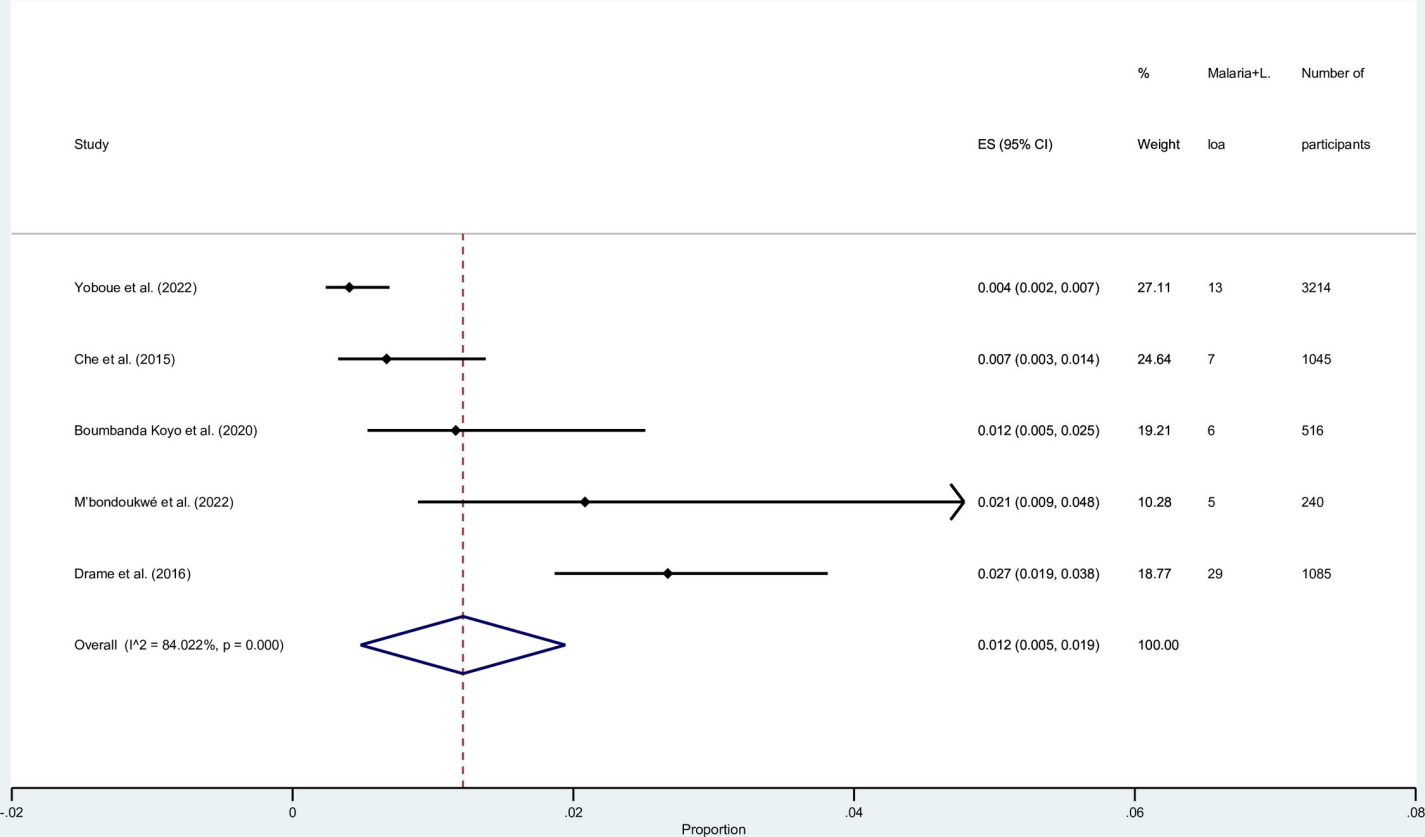
The prevalence of malaria and *L*. *loa* co-infections among selected studies. **Abbreviations**: ES- prevalence estimate; CI- confidence interval.

The pooled prevalence of malaria and *L*. *loa* co-infections among participants was estimated using the data available from five studies [24,26,28,32,38]. The results of the meta-analysis showed that participants’ pooled prevalence of malaria and *L*. *loa* co-infections was 1.2% (95% CI: 0.5%–1.9%, *I*^2^, 84%, 5 studies; Fig 5). The highest prevalence was demonstrated by the study conducted in Cameroon (2.7%, 95% CI: 1.9%–3.8%) [28], whereas the lowest prevalence was demonstrated by the study conducted in Equatorial Guinea (0.4%, 95% CI: 0.2%–0.7%) [38].

### Prevalence of malaria and *M*. *perstans* co-infections among selected studies

**Fig 6:**
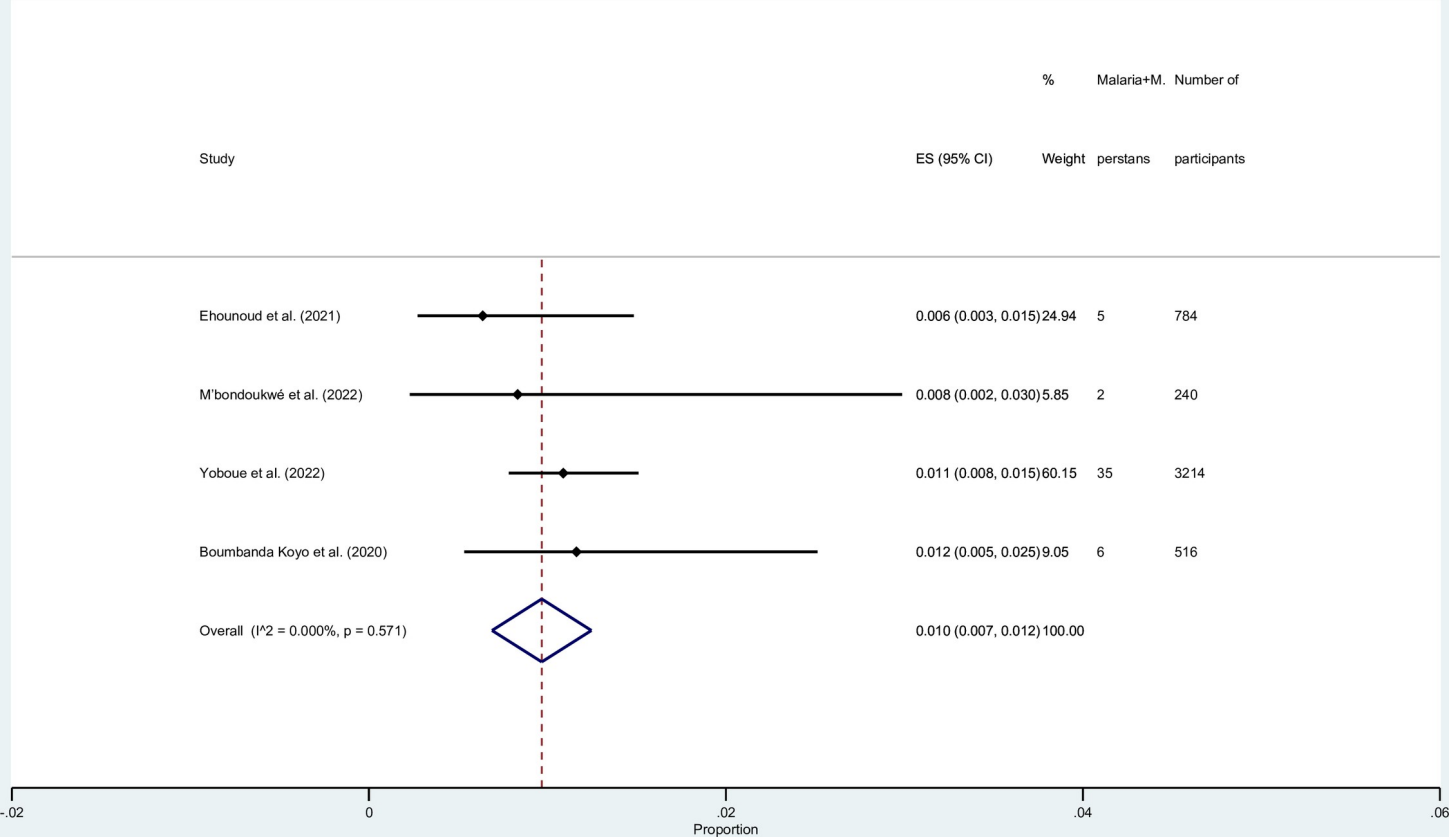
Prevalence of malaria and *M*. *perstans* co-infections among selected studies. **Abbreviations**: ES- prevalence estimate; CI-: confidence interval.

We estimated the pooled prevalence of malaria and *M*. *perstans* co-infections among participants using the data available from four studies [24,30,32,38]. The results of the meta-analysis showed that the pooled prevalence of malaria and *M*. *perstans* co-infections among participants was 1.0% (95% CI: 0.7%–1.2%, *I*^2^: 0%, 4 studies; Fig 6). The highest prevalence was demonstrated by the study conducted in Gabon (1.2%, 95% CI: 0.5%–2.5%) [24], whereas the lowest prevalence was demonstrated by the study conducted in Côte d’Ivoire (0.6%, 95% CI: 0.3%–1.5%) [30].

## Probability of co-infections among selected studies

**Fig 7:**
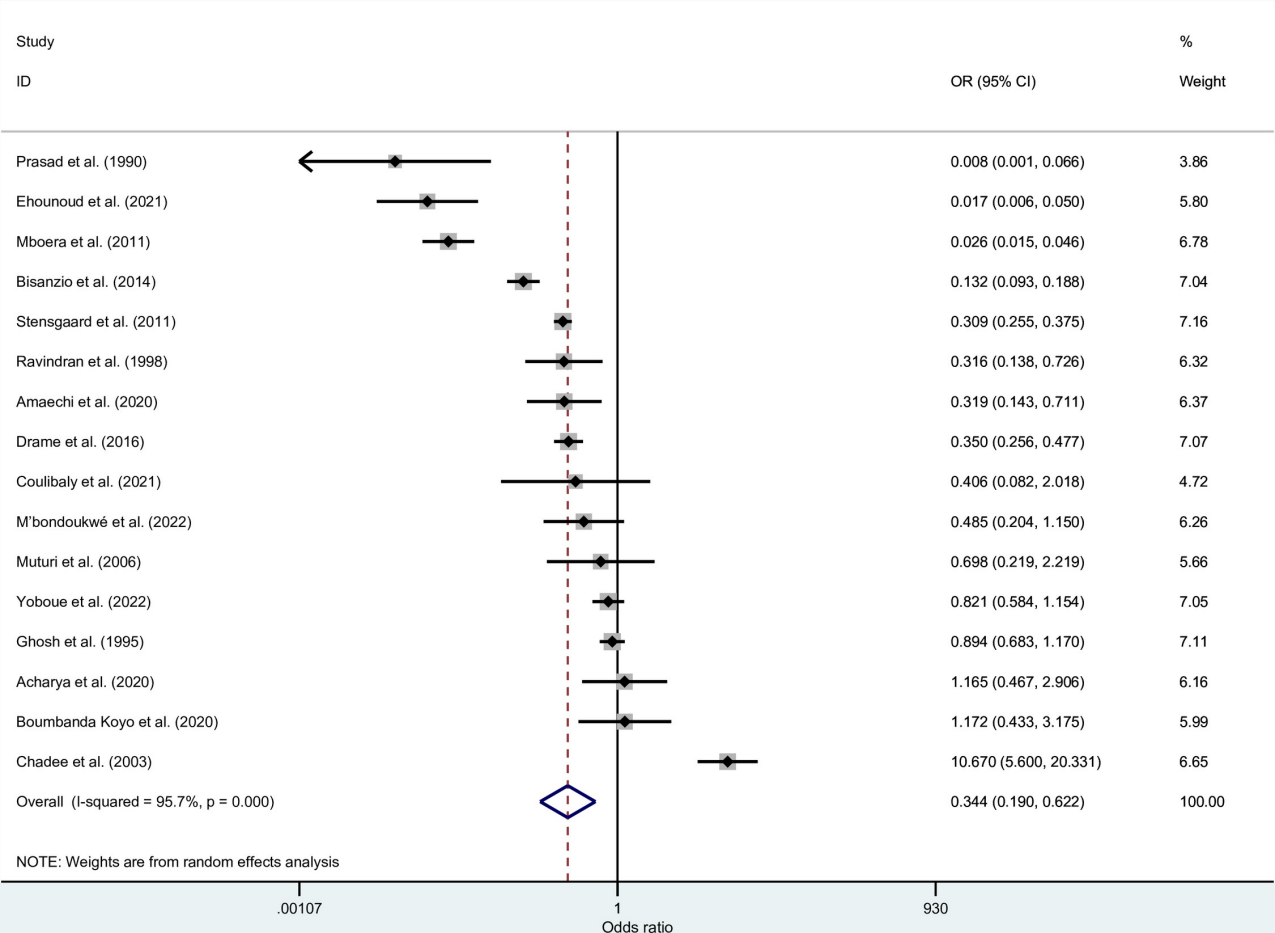
The odds of malaria and filariasis co-infections among selected studies. **Abbreviations**: OR- odds ratio; CI- confidence interval.

The probability of malaria and filariasis co-infections was estimated using the data available from 16 studies [22–25,27–38]. The results of the meta-analysis showed that the co-infections between the two parasites occurred by probability (*P* = 0.001, OR: 0.34, 95% CI: 0.19–0.62, *I*^2^: 95.7%, 16 studies; Fig 7).

Subgroup analysis of the probability of co-infections demonstrated that the likelihood of co-infections (*P* < 0.05) depended on continents, study design, participants, febrile conditions, age groups, diagnostic methods for malaria, and diagnostic methods for filariasis (Table 4). The probability of co-infections was low in all subgroup analyses (OR < 1).

**Table 4:** Subgroup analysis of the probability of co-infections among selected studies.

Subgroup	*P* value	Odds ratio	95% CI (%)	*I* ^2^	Number of studies
**Continents**					
Africa	< 0.001	0.25	0.14–0.45	94.0	11
Asia	0.074	0.35	0.11–1.11	87.9	4
South America	NA	10.7	5.60–20.3	NA	1
**Study design**					
Cross-sectional studies	< 0.001	0.22	0.13–0.39	94.8	13
Prospective observational studies	0.755	3.61	0.41–31.6	93.4	2
Retrospective observational studies	0.246	1.17	0.43–3.18	NA	1
**Participants**					
Participants in communities	< 0.001	0.27	0.16–0.47	94.5	12
Participants in hospitals/clinics	0.800	0.72	0.05–9.50	97	4
**Febrile conditions**					
Febrile	0.340	2.23	0.43–11.5	95.9	3
Febrile and afebrile	0.360	0.14	0–9–03	96.8	2
Not specified	< 0.001	0.24	0.14–0.42	93	11
**Age groups**					
All age groups	0.007	0.39	0.20–0.77	94.8	13
Children	0.095	0.21	0.03–1.32	97.4	3
**Diagnostic methods for malaria**					
Microscopy	0.027	0.34	0.13–0.89	96.8	9
Molecular method	0.092	0.20	0.03–1.31	94.3	3
Microscopy/RDT	0.743	1.17	0.47–2.91	NA	1
RDT/molecular method	0.224	0.33	0.06–1.97	98.1	2
Microscopy/molecular method	0.101	0.49	0.20–1.15	NA	1
**Diagnostic methods for filariasis**					
Microscopy	0.410	0.63	0.21–1.90	92.7	7
Molecular method	0.029	0.30	0.10–0.88	94.3	4
Direct microscopy/Leukoconcentration technique	0.101	0.49	0.20–1.15	NA	1
RDT	< 0.001	0.21	0.09–0.47	94.3	2
Microscopy/RDT	0.053	0.09	0–1.04	96	2

**Abbreviation:** CI- confidence interval; NA- not assessed; RDT- rapid diagnostic test

### Characteristics of co-infections among selected studies

Eight studies reported the characteristics of co-infections of malaria and filariasis co-infections [19–21,26,32,33,39,40]. Four of the eight studies (50%) compared the cytokine levels between co-infections and malaria monoinfection. Che et al. [26] enrolled febrile and afebrile participants in communities of Cameroon. They demonstrated that malaria and *L*. *loa* co-infections had decreased levels of C-X-C motif chemokine 5 (CXCL5) and comparable levels of CX3CL1, CXCL7, CXCL9, CXCL11, and CCL28 compared with malaria monoinfection. Olaniyan et al. [21] conducted a case-control study in Nigeria. They showed significantly lower plasma tumor necrosis factor–α, interleukin (IL)–4, and C4 levels in patients with co-infections compared with those with malaria monoinfection. M’bondoukwé et al. conducted a study in Gabon [32] and found that IL-10 was lower in patients with co-infections (median 224.5 pg/mL) compared with those who had malaria monoinfection (median 18.1 pg/mL). The IL-10/tumor necrosis factor–α ratio was also lower in patients with co-infections (10-fold) compared with those who had malaria monoinfection (30-fold). The IL-10/IL-6 ratio tended to be higher in patients with co-infections (twofold) compared with malaria monoinfection. Similarly, patients with co-infections had higher frequencies of CD4+ T cells producing IL-17A, IL-10, and IL-4 [20].

Four of the eight studies (50%) reported differences in clinical and laboratory characteristics between patients with co-infections and those with malaria monoinfection. Dolo et al. [19] performed a cohort study in Mali and reported no significant difference between filariasis-positive and filariasis-negative groups in the rate of clinical malaria. Mboera et al. [33], in a study that enrolled school children in Tanzania, showed a significantly higher parasite density among children with co-infections compared with those with monoinfection. In addition, there was an increased risk of having an enlarged spleen (4.6-fold) among children with co-infections. Moutongo Mouandza et al. [39] showed that patients with co-infections had a lower rate of severe anemia (44.4%) as compared with those with malaria monoinfection (69.5%). Treeprasertsuk et al. [40] reported that all patients with co-infections in Thailand were asymptomatic for filariasis and responded well to treatment.

### Publication bias

**Fig 8:**
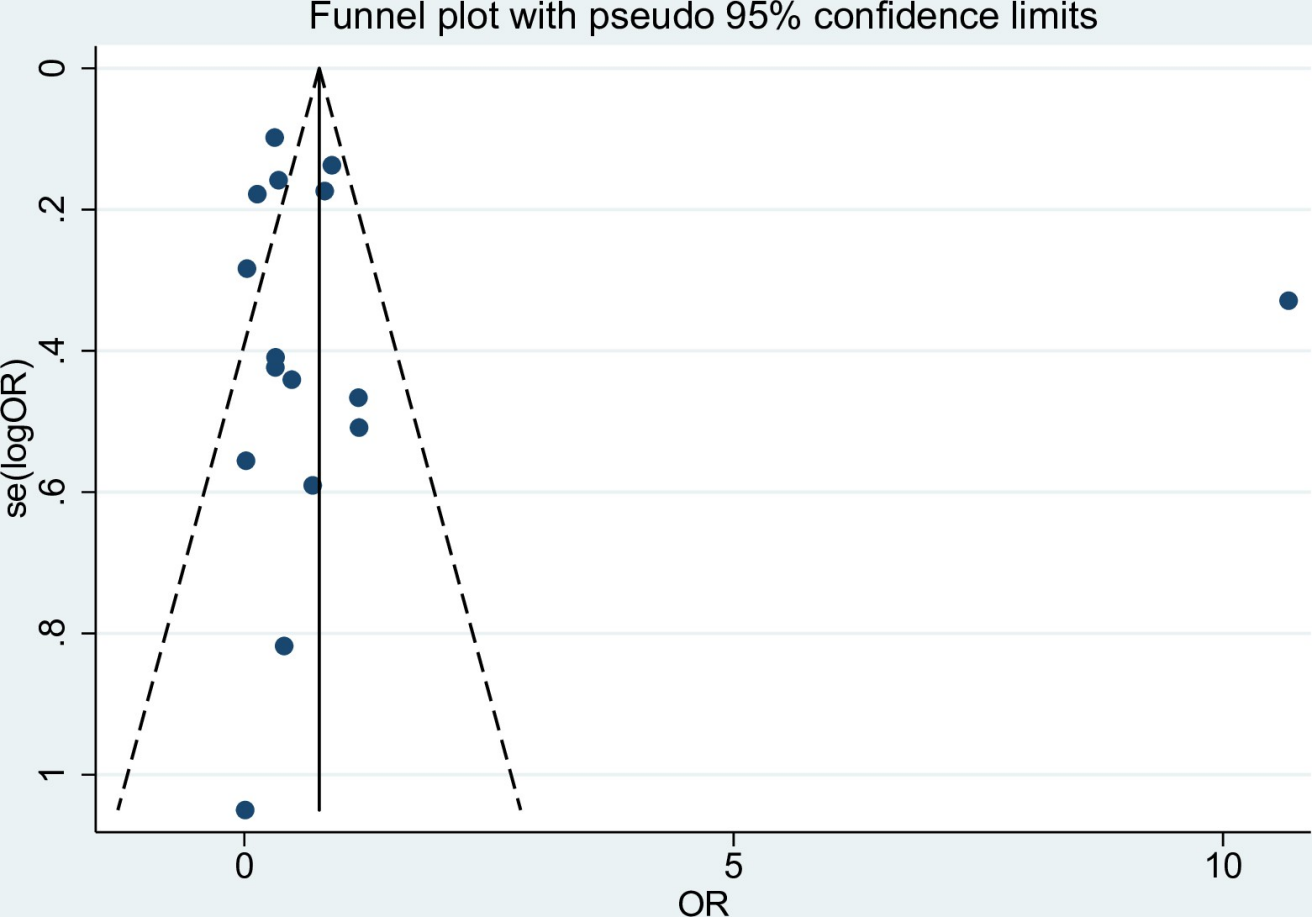
The funnel plot demonstrated the asymmetrical distribution of the effect estimate from the middle line (pooled OR). **Abbreviations**: OR- odds ratio.

**Fig 9:**
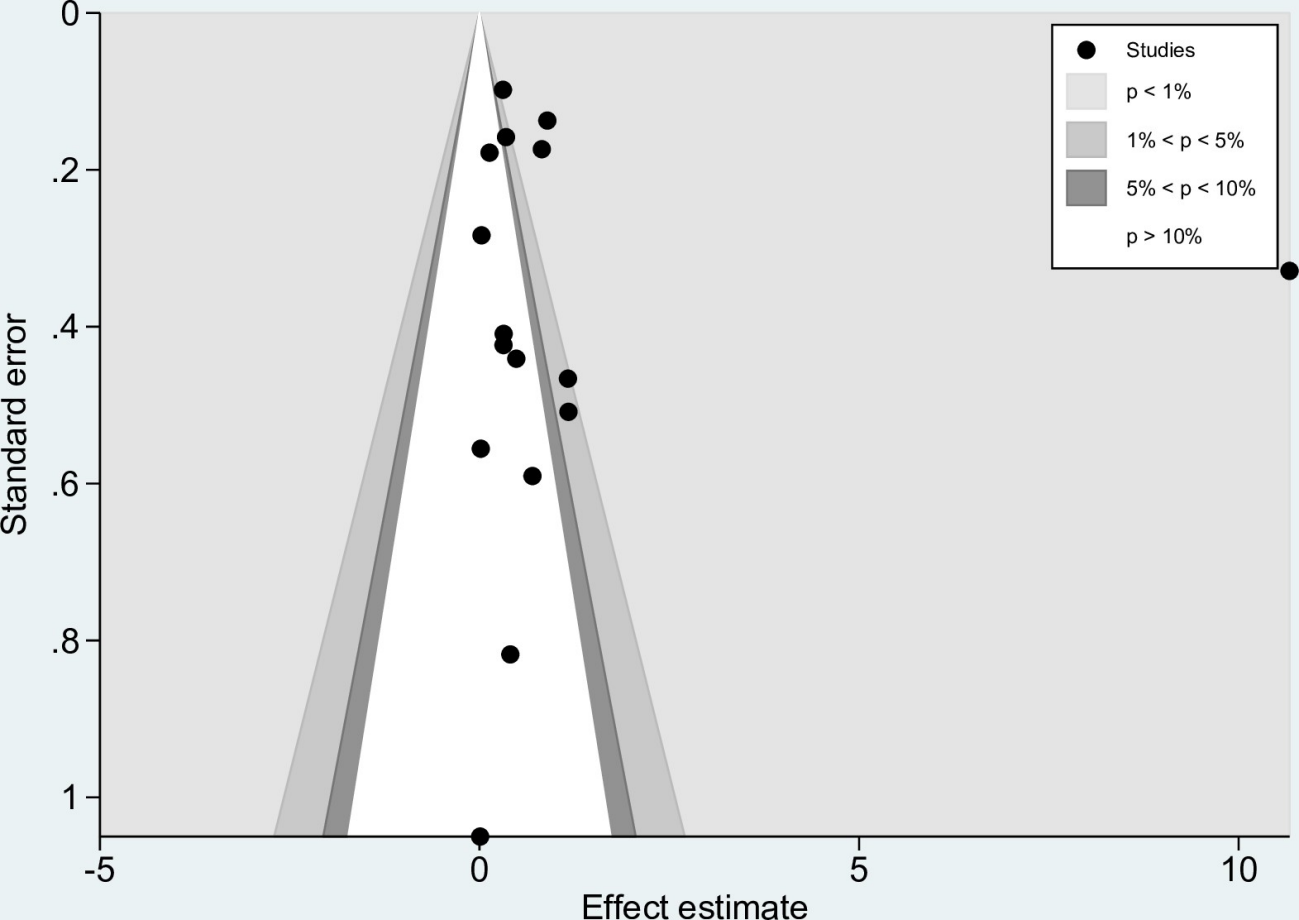
The contour-enhanced funnel plot demonstrated that the effect estimates were in both significant (*P* < 1%, 1% < *P* < 5%) and non-significant areas (5% < *P* < 10%, *P* > 10%).

We assessed the publication bias of the probability of co-infections by visualizing the funnel plot asymmetry and using Egger’s test. The funnel plot demonstrated the asymmetrical distribution of the effect estimate from the middle line (pooled OR; Fig 8). The Egger’s test showed no small-study effect (*P* < 0.455). The contour-enhanced funnel plot demonstrated that the effect estimates were in both significant and nonsignificant areas (Fig 9). Therefore, the asymmetry of the funnel plot might be caused by the heterogeneity of the probability of co-infections rather than publication bias.

## Discussion

In this study, we found that the participant-pooled prevalence of malaria and filariasis co-infections was very low at 0.7%. Nevertheless, the prevalence of this co-infection varied with several factors, such as geographical location, study design, participants, febrile condition, age group, diagnostic method for malaria or filariasis, and different filarial parasites. Therefore, it is postulated that the rate of malaria and filariasis co-infections may be higher in areas where both diseases are highly endemic. This hypothesis was supported by the subgroup analysis, which demonstrated a higher prevalence of co-infections in Africa (2.2%) than in Asia (0.2%). The highest prevalence of co-infections by country included Cameroon (12.7%) [28], India (13.1%) [35], and Tanzania (14%). *M*. *perstans* (76%), *L*. *loa* (39%), and *P*. *falciparum* (33%) co-infection was reported in Cameroon. Another study in Tanzania that demonstrated the highest rate of co-infections (14%) suggested that the co-infections were caused by the availability of the mosquito vectors in the area, including *An*. *gambiae* and *An*. *funestus*. Therefore, the prevalence of malaria and filariasis co-infections might be high according to the high prevalence of both diseases with the abundance of the vectors in the same area.

The meta-analysis of the prevalence of co-infection by filarial parasites showed that the pooled prevalence of malaria and *W*. *bancrofti* co-infections among participants was similar to the overall prevalence of co-infections (0.7%). However, a higher prevalence of co-infections was reported with *M*. *perstans* (1.0%) and *L*. *loa* co-infections (1.2%). The low prevalence of malaria and *W*. *bancrofti* co-infections could be attributed to the difficulty in detecting *W*. *bancrofti* in blood smear because of its nocturnal subperiodic nature of circulation, particularly in low parasitemia [22]. In addition, most cases of Bancroftian filariasis were asymptomatic [41]; therefore, the exact prevalence of the co-infection might be higher than previously observed. A previous study showed that *M*. *perstans* was often asymptomatic and was detected in only febrile patients; meanwhile, *L*. *loa* was present more often in both febrile and afebrile individuals [24].

The distribution of concomitant infections may be attributable to differences in behavior and occupation between age groups. In their study, Bisanzio et al. found that filariasis and hookworms mostly affected adults, but malaria, schistosomiasis, and *Trichuris* mostly affected patients aged 8 to 16 years [23]. This finding is similar to another study that reported that co-infection was less prevalent among those aged 0 to 9 years [25]. Adults aged between 30 and 49 years are more likely to work outdoors than indoors, and potent vectors for both malaria or filariasis may be more exophily, which might potentiate increased vector–human contact and increase transmission among this age group. Similarly, males tend to acquire concomitant infections more often than females do, because males are at risk of mosquito bites while working outdoors, such as in mines and during other forest-related work, whereas females are more likely to work indoors [25].

Co-infections in malaria and other parasites were reported previously and occurred by chance in varied geographic regions [42]. The present study also found that co-infections of malaria and filariasis occurred by probability. It is known that the major *Anopheles* species can transmit both malaria and filariasis [11], but the distribution of these vectors vary by different geographical locations. In Africa, *An*. *gambiae*, *An*. *arabiensis*, *An*. *merus*, *An*. *merus*, and *An*. *funestus* are the major *Anopheles* species that transmit malaria and filariasis. However, *An*. *philippinensis* and *An*. *barbirostris* are the main vectors for malaria and filariasis in India and Bangladesh [11]. There were reports about concomitant infections of the two parasites in the vector, but they occurred coincidentally and were rare [34,43,44]. The lower likelihood of co-infections suggested by meta-analysis may be attributable to the activation of the phenoloxidase cascade in response to microfilariae in the hemolymph, which can also be used against oocysts in the midgut [45]. Another reason was the existence of the degree of interspecies competition between two parasites to dominate the other within the vector or human host [46]. Therefore, it is possible that the reduced probability of malaria and filariasis co-infections was due to the low prevalence of both parasites [34,46]. In addition, mosquitoes harboring low microfilariae densities might survive longer and increase their chances of ingesting malaria parasites [47]. Muturi et al. reported a significantly higher rate of sporozoites in *An*. *gambiae* infected with *Wuchereria* than in noninfected mosquitoes, indicating that *W*. *bancrofti* infection may increase mosquito susceptibility to *P*. *falciparum* infection [34]. Another review explained that *Anopheles* species had higher *P*. *falciparum* infection rates than those of *W*. *bancrofti* and thus described the more extended latent period of *W*. *bancrofti* in the vector [48].

As for our qualitative synthesis, distinct clinical characteristics of patients with co-infections compared with those with malaria monoinfection were at increased risk of having an enlarged spleen, lower rate of severe anemia, and more likely to be asymptomatic. Moutongo Mouandza et al. suggested that co-infections may protect patients from anemia by reducing the contribution of the inflammatory immune response [39]. Distinct laboratory data included a higher parasite density in patients with co-infections than in those with malaria monoinfection. Ghosh et al. showed that the density of *P*. *falciparum* parasite was lower in those with co-infection than in those without filariasis [31], and Muturi et al. suggested that filarial infections might protect against the development of malaria [11]. Finally, there were distinct cytokine profiles in patients with co-infections compared with those with malaria monoinfections. In vitro stimulations of cytokine response suggested that patients with malaria concomitant with filarial infection may lose protection against severe malaria [20]. However, because most studies included in the analysis included cross-sectional studies, it was difficult to deduce the above conclusion.

The present study had some limitations. First, the number of included studies was low; however, the results of pooling data from these studies would help provide the current status of the diseases and may indicate the interference of malaria on eliminating filariasis. Second, the characteristics of malaria and filariasis co-infections were also limited because of the limited investigations of these parasites’ co-infection in the literature. Limitations on the details of the cytokine levels in co-infections were based on one-off studies. Even though these are important observations, it may not be prudent to generalize the cytokine expression levels in co-infections based on these studies. In addition, the population size was greatly reduced and insufficient for generalization, for example, for a continent. Third, there was heterogeneity in the pooled prevalence and probability of co-infections among participants. Therefore, careful interpretation of the pooled prevalence of co-infections is recommended. Fourth, because publication bias among the studies included in the meta-analysis of co-infection was less likely, we did not perform the trim-and-fill method to correct the pooled effect estimate.

## Conclusion

The present study showed that the prevalence of malaria and filariasis co-infections was low and varied between geographical areas in the selected articles. Co-infections tended to occur with a low probability. Because few details on the characteristics of malaria and filariasis co-infections have been reported in the literature, further studies investigating the outcomes and characteristics of co-infections are needed if co-infections become a health problem.

## Supporting information

S1 PRISMA Abstract Checklist Prevalence, probability, and characteristics of malaria and filariasis co-infections: A systematic review and meta-analysis. (DOCX)

S2 PRISMA 2020 Checklist Prevalence, probability, and characteristics of malaria and filariasis co-infections: A systematic review and meta-analysis. (DOCX)

S1 Table Search terms. (DOCX)

S2 Table Details of the included studies. (XLSX)

S3 Table Quality of the included studies. (DOCX)

## References

[1] Mahittikorn A, Masangkay FR, Kotepui KU, Milanez GJ, Kotepui M. Comparison of *Plasmodium ovale curtisi* and *Plasmodium ovale wallikeri* infections by a meta-analysis approach. Sci Rep. 2021;11(1):6409. 33742015 10.1038/s41598-021-85398-w PMC7979700

[2] WHO. World malaria report 2021 [cited 2022 20 June]. Available from: https://www.who.int/teams/global-malaria-programme/reports/world-malaria-report-2021.

[3] Taylor MJ, Hoerauf A, Bockarie M. Lymphatic filariasis and onchocerciasis. Lancet. 2010;376(9747):1175–85. doi: 10.1016/S0140-6736(10)60586-7 20739055

[4] Wynd S, Melrose WD, Durrheim DN, Carron J, Gyapong M. Understanding the community impact of lymphatic filariasis: a review of the sociocultural literature. Bull World Health Organ. 2007;85(6):493–8. doi: 10.2471/blt.06.031047 17639248 PMC2636343

[5] Cano J, Basanez MG, O’Hanlon SJ, Tekle AH, Wanji S, Zoure HG, et al. Identifying co-endemic areas for major filarial infections in sub-Saharan Africa: seeking synergies and preventing severe adverse events during mass drug administration campaigns. Parasit Vectors. 2018;11(1):70. doi: 10.1186/s13071-018-2655-5 29382363 PMC5791223

[6] Kao JH, Chen CD, Tiger Li ZR, Chan TC, Tung TH, Chu YH, et al. The critical role of early dengue surveillance and limitations of clinical reporting—implications for non-endemic countries. PLoS One. 2016;11(8):e0160230. doi: 10.1371/journal.pone.0160230 27501302 PMC4976904

[7] WHO. Lymphatic filariasis: WHO; 2022 [cited 2022 2 September]. Available from: https://www.who.int/news-room/fact-sheets/detail/lymphatic-filariasis.

[8] Local Burden of Disease 2019 Neglected Tropical Diseases Collaborators. The global distribution of lymphatic filariasis, 2000–18: a geospatial analysis. Lancet Glob Health. 2020;8(9):e1186–e94. doi: 10.1016/S2214-109X(20)30286-2 32827480 PMC7443698

[9] CDC. Parasites—Loiasis: CDC; 2020 [cited 2022 23 June]. Available from: https://www.cdc.gov/parasites/loiasis/index.html.

[10] CDC. Mansonellosis 2019 [cited 2022 23 June]. Available from: https://www.cdc.gov/dpdx/mansonellosis/index.html.

[11] Muturi EJ, Jacob BG, Kim CH, Mbogo CM, Novak RJ. Are coinfections of malaria and filariasis of any epidemiological significance? Parasitol Res. 2008;102(2):175–81. doi: 10.1007/s00436-007-0779-1 18026992

[12] WHO. Integrated vector management (IVM) WHO 2022 [cited 2022 3 September]. Available from: https://www.who.int/europe/news-room/fact-sheets/item/integrated-vector-management-(ivm).

[13] Shen SS, Qu XY, Zhang WZ, Li J, Lv ZY. Infection against infection: parasite antagonism against parasites, viruses and bacteria. Infect Dis Poverty. 2019;8(1):49. doi: 10.1186/s40249-019-0560-6 31200765 PMC6570864

[14] Page MJ, McKenzie JE, Bossuyt PM, Boutron I, Hoffmann TC, Mulrow CD, et al. The PRISMA 2020 statement: an updated guideline for reporting systematic reviews. BMJ. 2021;372:n71. doi: 10.1136/bmj.n71 33782057 PMC8005924

[15] Moola S, Munn Z, Tufanaru C, Aromataris E, Sears K, Sfetcu R, Currie M, Qureshi R, Mattis P, Lisy K, Mu P-F. Chapter 7: Systematic reviews of etiology and risk. Aromataris E, Munn Z (Editors): The Joanna Briggs Institute; 2017. 10.1097/XEB.0000000000000064 26262566

[16] Mahittikorn A, Mala W, Srisuphanunt M, Masangkay FR, Kotepui KU, Wilairatana P, et al. Tumour necrosis factor-alpha as a prognostic biomarker of. severe malaria: a systematic review and meta-analysis. J Travel Med. 2022;29(4):taac053 35467747 10.1093/jtm/taac053

[17] Wilairatana P, Mala W, Milanez GJ, Masangkay FR, Kotepui KU, Kotepui M. Increased interleukin-6 levels associated with malaria infection and disease severity: a systematic review and meta-analysis. Sci Rep. 2022;12(1):5982. doi: 10.1038/s41598-022-09848-9 35396564 PMC8993930

[18] DerSimonian R, Laird N. Meta-analysis in clinical trials revisited. Contemp Clin Trials. 2015;45(Pt A):139–45. doi: 10.1016/j.cct.2015.09.002 26343745 PMC4639420

[19] Dolo H, Coulibaly YI, Dembele B, Konate S, Coulibaly SY, Doumbia SS, et al. Filariasis attenuates anemia and proinflammatory responses associated with clinical malaria: a matched prospective study in children and young adults. PLoS Negl Trop Dis. 2012;6(11):e1890. doi: 10.1371/journal.pntd.0001890 23133692 PMC3486872

[20] Metenou S, Dembele B, Konate S, Dolo H, Coulibaly YI, Diallo AA, et al. Filarial infection suppresses malaria-specific multifunctional Th1 and Th17 responses in malaria and filarial coinfections. J Immunol. 2011;186(8):4725–33. doi: 10.4049/jimmunol.1003778 21411732 PMC3407819

[21] Olaniyan MF, Ojo KO, Azeez MM, Afolabi T. Plasma complement 4, tumor necrosis factor-alpha, and interleukin 4 in patients co-infected with *Plasmodium* coinfection and filarial worm. Imam Journal of Applied Sciences. 2018;3(2):63–7.

[22] Acharya A, Rakshit A, Halder S, Chatterjee M, Chakrabarti S, Saha P, et al. Coexistent malaria and filaria among the febrile patients attending for malaria diagnosis: A clinic-based study. Trop Parasitol. 2020;10(2):109–13. doi: 10.4103/tp.TP_93_20 33747877 PMC7951078

[23] Bisanzio D, Mutuku F, Bustinduy AL, Mungai PL, Muchiri EM, King CH, et al. Cross-sectional study of the burden of vector-borne and soil-transmitted polyparasitism in rural communities of Coast Province, Kenya. PLoS Negl Trop Dis. 2014;8(7):e2992. doi: 10.1371/journal.pntd.0002992 25057825 PMC4109907

[24] Boumbanda Koyo CS, Oyegue-Liabagui SL, Mediannikov O, Cortaredona S, Kouna LC, Raoult D, et al. High circulation of malaria and low prevalence of bacteremia in febrile and afebrile children in northeastern Gabon. Am J Trop Med Hyg. 2020;102(1):121–9. doi: 10.4269/ajtmh.19-0368 31769404 PMC6947801

[25] Chadee DD, Rawlins SC, Tiwari TS. Short communication: concomitant malaria and filariasis infections in Georgetown, Guyana. Trop Med Int Health. 2003;8(2):140–3. doi: 10.1046/j.1365-3156.2003.01001.x 12581439

[26] Che JN, Nmorsi OP, Nkot BP, Isaac C, Okonkwo BC. Chemokines responses to *Plasmodium falciparum* malaria and co-infections among rural Cameroonians. Parasitol Int. 2015;64(2):139–44. 25462711 10.1016/j.parint.2014.11.003

[27] Coulibaly S, Sawadogo SP, Hien AS, Nikièma AS, Sangaré I, Rabila B, Koala L, Bougouma C, Windtaré Bougma R, Ouedraogo GA, Dabiré RK. Malaria and lymphatic filariasis co-transmission in endemic health districts in Burkina Faso. Adv Entomol. 2021;9:155–75.

[28] Drame PM, Montavon C, Pion SD, Kubofcik J, Fay MP, Nutman TB. Molecular epidemiology of blood-borne human parasites in a *Loa loa*-, *Mansonella perstans*-, and *Plasmodium falciparum*-endemic region of Cameroon. Am J Trop Med Hyg. 2016;94(6):1301–8. 27044568 10.4269/ajtmh.15-0746 PMC4889748

[29] Amaechi EC, Ohaer CC, Ukpai OM Co-infection of *Plasmodium falciparum* and Wuchereria bancrofti in an irrigated farming community, North Central Nigeria. Moroccan J Biol. 2020;17:43–6.

[30] Ehounoud BCH, Boumbanda Koyo CS, Doua Bongue L, Cortaredona S, N’Douba Kakou A, Konan DB, et al. Assessment of the burden of malaria and bacteraemia by retrospective molecular diagnosis in febrile illnesses and first-line anti-infectives in Côte d’Ivoire. Travel Med Infect Dis. 2021;43. doi: 10.1016/j.tmaid.2021.102105 34146685

[31] Ghosh SK, Yadav RS. Naturally acquired concomitant infections of bancroftian filariasis and human plasmodia in Orissa. Indian J Malariol. 1995;32(1):32–6. 8549837

[32] M’bondoukwé NP, Moutongo R, Gbédandé K, Ngomo JMN, Hountohotegbé T, Adamou R, et al. Circulating IL-6, IL-10, and TNF-alpha and IL-10/IL-6 and IL-10/TNF-alpha ratio profiles of polyparasitized individuals in rural and urban areas of gabon. PLoS Negl Trop Dis. 2022;16(4). 10.1371/journal.pntd.0010308 PMC9041759 35421083

[33] Mboera LEG, Senkoro KP, Rumisha SF, Mayala BK, Shayo EH, Mlozi MRS. *Plasmodium falciparum* and helminth coinfections among schoolchildren in relation to agro-ecosystems in Mvomero District, Tanzania. Acta Trop. 2011;120(1–2):95–102. 21741929 10.1016/j.actatropica.2011.06.007

[34] Muturi EJ, Mbogo CM, Mwangangi JM, Ng’ang’a ZW, Kabiru EW, Mwandawiro C, et al. Concomitant infections of *Plasmodium falciparum* and *Wuchereria bancrofti* on the Kenyan coast. Filaria J. 2006;5:8. 16723020 10.1186/1475-2883-5-8 PMC1513226

[35] Prasad RN, Prasad H. Prasad Sharma VP. Concomitant occurrence of malaria and filariasis in man in India. Mosquito Borne Diseases Bulletin. 1990;7(2):51–3.

[36] Ravindran B, Sahoo PK, Dash AP. Lymphatic filariasis and malaria: concomitant parasitism in Orissa, India. Trans R Soc Trop Med Hyg. 1998;92(1):21–3. doi: 10.1016/s0035-9203(98)90937-3 9692139

[37] Stensgaard A-S, Vounatsou P, Onapa AW, Simonsen PE, Pedersen EM, Rahbek C, et al. Bayesian geostatistical modelling of malaria and lymphatic filariasis infections in Uganda: predictors of risk and geographical patterns of co-endemicity. Malar J. 2011;10:298. doi: 10.1186/1475-2875-10-298 21989409 PMC3216645

[38] Yoboue CA, Hosch S, Donfack OT, Guirou EA, Nlavo BM, Ayekaba MO, et al. Characterising co-infections with *Plasmodium* spp., *Mansonella perstans* or *Loa loa* in asymptomatic children, adults and elderly people living on Bioko Island using nucleic acids extracted from malaria rapid diagnostic tests. PLoS Negl Trop Dis. 2022;16(1):e0009798. 35100277 10.1371/journal.pntd.0009798 PMC8830708

[39] Moutongo Mouandza R, M’Bondoukwe NP, Obiang Ndong GP, Nzaou Nziengui A, Batchy Ognagosso FB, Nziengui Tirogo C, et al. Anaemia in asymptomatic parasite carriers living in urban, rural and peri-urban settings of Gabon. Trans R Soc Trop Med Hyg. 2020;114(8):618–26. doi: 10.1093/trstmh/traa047 32609837

[40] Treeprasertsuk S, Chindanond D, Wilairatana P, Krudsood S, Bussaratid V, Glanarongran R, Srinukham S. Incidence of filariasis as a co-infection in malaria patients coming from Thai-Myanmar border between 1995–1997. Jpn J Trop Med Hyg. 1998;26(4):323–6.

[41] Kar SK, Dwibedi B, Das BK, Agrawala BK, Ramachandran CP, Horton J. Lymphatic pathology in asymptomatic and symptomatic children with *Wuchereria bancrofti* infection in children from Odisha, India and its reversal with DEC and albendazole treatment. PLoS Negl Trop Dis. 2017;11(10):e0005631. 29059186 10.1371/journal.pntd.0005631 PMC5667936

[42] Mahittikorn A, Masangkay FR, De Jesus Milanez G, Kuraeiad S, Kotepui M. Prevalence and effect of *Plasmodium* spp. and hookworm co-infection on malaria parasite density and haemoglobin level: a meta-analysis. Sci Rep. 2022;12(1):6864. 35477943 10.1038/s41598-022-10569-2 PMC9046215

[43] Muirhead-Thomson RC. Inter-relations between filarial and malarial infections in *Anopheles gambiae*. Nature. 1953;172(4372):352–3. 13087232 10.1038/172352a0

[44] Bockarie MJ, Tavul L, Kastens W, Michael E, Kazura JW. Impact of untreated bednets on prevalence of *Wuchereria bancrofti* transmitted by *Anopheles farauti* in Papua New Guinea. Med Vet Entomol. 2002;16(1):116–9. Epub 2002/04/20. doi: 10.1046/j.0269-283x.2002.00352.x . 11963977

[45] Albuquerque CM, Ham PJ. Concomitant malaria (*Plasmodium gallinaceum*) and filaria (Brugia pahangi) infections in *Aedes aegypti*: effect on parasite development. Parasitology. 1995;110 (Pt 1):1–6. 7845706 10.1017/s0031182000080987

[46] Kelly-Hope LA, Diggle PJ, Rowlingson BS, Gyapong JO, Kyelem D, Coleman M, et al. Short communication: Negative spatial association between lymphatic filariasis and malaria in West Africa. Trop Med Int Health. 2006;11(2):129–35. doi: 10.1111/j.1365-3156.2005.01558.x 16451336

[47] Klein TA, Harrison BA, Grove JS, Dixon SV, Andre RG. Correlation of survival rates of *Anopheles dirus* A (Diptera: Culicidae) with different infection densities of *Plasmodium cynomolgi*. Bull World Health Organ. 1986;64(6):901–7. 3493859 PMC2490972

[48] Manguin S, Bangs MJ, Pothikasikorn J, Chareonviriyaphap T. Review on global co-transmission of human *Plasmodium* species and *Wuchereria bancrofti* by *Anopheles* mosquitoes. Infect Genet Evol. 2010;10(2):159–77. 19941975 10.1016/j.meegid.2009.11.014

