# Supplementary material for: Prevalence, probability, and characteristics of malaria and filariasis co-infections: A systematic review and meta-analysis
Source: PLoS Negl Trop Dis. 2022 Oct 21;16(10):e0010857. doi: 10.1371/journal.pntd.0010857 (PMC9586402; doi:10.1371/journal.pntd.0010857)
Supplement: S1 Table — (DOCX) [file pntd.0010857.s003.docx]

**Prevalence, probability, and characteristics of malaria and filariasis co-infection**s**: A systematic review and meta-analysis**

Polrat Wilairatana^1^, Kwuntida Uthaisar Kotepui^2^, Wanida Mala^2^, Kinley Wangdi^3^, Manas Kotepui^2*^

^1^Department of Clinical Tropical Medicine, Faculty of Tropical Medicine, Mahidol University, Bangkok, Thailand; polrat.wil@mahidol.ac.th (P.W.)

^2^Medical Technology, School of Allied Health Sciences, Walailak University, Tha Sala, Nakhon Si Thammarat, Thailand; kwuntida.ut@wu.ac.th (K.U.K.), wanida.ma@wu.ac.th (W.M.), manas.ko@wu.ac.th (M.K.)

^3^Department of Global Health, National Centre for Epidemiology and Population Health, College of Health & Medicine, Australian National University, Canberra, Acton, ACT 2601, Australia; kinley.wangdi@anu.edu.au (K.W.)

*****Correspondence Author: Manas Kotepui; manas.ko@wu.ac.th (M.K.)

**Table S1. Search strategy**

**Embase**

**22 May 2022**

| No. | Query | Results |
| --- | --- | --- |
| #4 | #1 AND #2 AND #3 | 331 |
| #3 | coinfect* OR 'co infect*' OR concurent* OR mix* OR 'co occur*' OR coincident OR coincidental OR coinciding OR cooccur* OR simultaneous | 1465861 |
| #2 | 'malaria'/exp OR malaria OR 'plasmodium'/exp OR plasmodium | 155696 |
| #1 | filaria* OR elephantias* OR 'lymphoedema'/exp OR lymphoedema OR 'lymphadenopathy'/exp OR lymphadenopathy OR 'acute dermatolymphangioadenit*' OR adla OR lymphangit* | 345236 |

**PubMed**

**22 May 2022**

| Search number | Query | Search Details | Results |
| --- | --- | --- | --- |
| 3 | #1 AND #2 | ("filaria"[Title/Abstract] OR "filaria"[Text Word] OR "elephantiasis"[MeSH Terms] OR "elephantiasis"[Title/Abstract] OR "elephantiasis"[Text Word] OR "lymphedema"[MeSH Terms] OR "lymphoedema"[Title/Abstract] OR "lymphoedema"[Text Word] OR "lymphadenopathy"[MeSH Terms] OR "lymphadenopathy"[Title/Abstract] OR "lymphadenopathy"[Text Word] OR "Lymphangitis"[MeSH Terms] OR "Lymphangitis"[Title/Abstract] OR "Lymphangitis"[Text Word]) AND ("malaria"[MeSH Terms] OR "malaria"[Title/Abstract] OR "malaria"[Text Word] OR "plasmodium"[MeSH Terms] OR "plasmodium"[Title/Abstract] OR "plasmodium"[Text Word]) | 268 |
| 2 | (((((malaria[MeSH Terms]) OR (malaria[Title/Abstract])) OR (malaria[Text Word])) OR (plasmodium[MeSH Terms])) OR (plasmodium[Title/Abstract])) OR (plasmodium[Text Word]) | "malaria"[MeSH Terms] OR "malaria"[Title/Abstract] OR "malaria"[Text Word] OR "plasmodium"[MeSH Terms] OR "plasmodium"[Title/Abstract] OR "plasmodium"[Text Word] | 113,436 |
| 1 | (((((((((((((((((filaria[MeSH Terms]) OR (filaria[Title/Abstract])) OR (filaria[Text Word])) OR (elephantiasis[MeSH Terms])) OR (elephantiasis[Title/Abstract])) OR (elephantiasis[Text Word])) OR (lymphoedema[MeSH Terms])) OR (lymphoedema[Title/Abstract])) OR (lymphoedema[Text Word])) OR (lymphadenopathy[MeSH Terms])) OR (lymphadenopathy[Title/Abstract])) OR (lymphadenopathy[Text Word])) OR (Acute dermatolymphangioadenit[MeSH Terms])) OR (Acute dermatolymphangioadenit[Title/Abstract])) OR (Acute dermatolymphangioadenit[Text Word])) OR (Lymphangitis[MeSH Terms])) OR (Lymphangitis[Title/Abstract])) OR (Lymphangitis[Text Word]) | "filaria"[Title/Abstract] OR "filaria"[Text Word] OR "elephantiasis"[MeSH Terms] OR "elephantiasis"[Title/Abstract] OR "elephantiasis"[Text Word] OR "lymphedema"[MeSH Terms] OR "lymphoedema"[Title/Abstract] OR "lymphoedema"[Text Word] OR "lymphadenopathy"[MeSH Terms] OR "lymphadenopathy"[Title/Abstract] OR "lymphadenopathy"[Text Word] OR "Lymphangitis"[MeSH Terms] OR "Lymphangitis"[Title/Abstract] OR "Lymphangitis"[Text Word] | 42,487 |

**CENTRAL**

**22 May 2022**

| **ID** | **Search Hits** | **Results** |
| --- | --- | --- |
| #1 | ((filaria* OR elephantias* OR lymphoedema OR lymphadenopathy OR "Acute dermatolymphangioadenit*" OR ADLA OR lymphangit*)):ti,ab,kw (Word variations have been searched) | 2793 |
| #2 | ((malaria OR plasmodium)):ti,ab,kw (Word variations have been searched) | 7146 |
| #3 | MeSH descriptor: [Malaria] explode all trees | 3291 |
| #4 | MeSH descriptor: [Filariasis] explode all trees | 350 |
| #5 | #1 OR #4 | 2941 |
| #6 | #2 OR #3 | 7146 |
| #7 | #5 AND #6 | 35 |

| **Databases** | **Search terms/Search strategy** | **Date** |
| --- | --- | --- |
| MEDLINE | (filaria* OR elephantias* OR lymphoedema OR lymphadenopathy OR "Acute dermatolymphangioadenit*" OR ADLA OR lymphangit*) AND (malaria OR plasmodium) AND (coinfect* OR co-infect* OR concurent* OR mix* OR co-occur* OR coincident OR coincidental OR coinciding OR cooccur* OR simultaneous)  Search results: 135 | 22 May 2022 |
| Scopus | (filaria* OR elephantias* OR lymphoedema OR lymphadenopathy OR "Acute dermatolymphangioadenit*" OR ADLA OR lymphangit*) AND (malaria OR plasmodium) AND (coinfect* OR co-infect* OR concurent* OR mix* OR co-occur* OR coincident OR coincidental OR coinciding OR cooccur* OR simultaneous)  Search option: Title, abstract, keywords  Search results: 182 | 22 May 2022 |
