# Supplementary material for: Prevalence, probability, and characteristics of malaria and filariasis co-infections: A systematic review and meta-analysis
Source: PLoS Negl Trop Dis. 2022 Oct 21;16(10):e0010857. doi: 10.1371/journal.pntd.0010857 (PMC9586402; doi:10.1371/journal.pntd.0010857)
Supplement: S3 Table — (DOCX) [file pntd.0010857.s005.docx]

**Table S3. Quality of the included studies**

**Cohort studies**

| **No.** | **Study** | **1. Were the two groups similar and recruited from the same population?** | **2. Were the exposures measured similarly to assign people to both exposed and unexposed groups?** | **3. Was the exposure measured in a valid and reliable way?** | **4. Were confounding factors identified?** | **5. Were strategies to deal with confounding factors stated?** | **6. Were the groups/participants free of the outcome at the start of the study (or at the moment of exposure)?** | **7. Were the outcomes measured in a valid and reliable way?** | **8. Was the follow up time reported and sufficient to be long enough for outcomes to occur?** | **9. Was follow up complete, and if not, were the reasons to loss to follow up described and explored?** | **10. Were strategies to address incomplete follow up utilized?** | **11. Was appropriate statistical analysis used?** | **Score (percentile)** | **Overall appraisal:** |
| --- | --- | --- | --- | --- | --- | --- | --- | --- | --- | --- | --- | --- | --- | --- |
| 1 | Dolo et al. (2012) | Yes | Yes | Yes | Yes | Yes | Yes | Yes | Yes | Yes | No | Yes | 10 (90.9) | Included |
| 2 | Metenou et al. (2011) | Yes | Yes | Yes | Yes | Yes | Yes | Yes | Unclear | Unclear | Unclear | Yes | 8 (72.7) | Included |

**Case-control studies**

| **No.** | **Study** | **1. Were the groups comparable other than the presence of disease in cases or the absence of disease in controls?** | **2. Were cases and controls matched appropriately?** | **3. Were the same criteria used for identification of cases and controls?** | **4. Was exposure measured in a standard, valid and reliable way?** | **5. Was exposure measured in the same way for cases and controls?** | **6. Were confounding factors identified?** | **7. Were strategies to deal with confounding factors stated?** | **8. Were outcomes assessed in a standard, valid and reliable way for cases and controls?** | **9. Was the exposure period of interest long enough to be meaningful?** | **10. Was appropriate statistical analysis used?** | **Score (percentile)** | **Overall appraisal:** |
| --- | --- | --- | --- | --- | --- | --- | --- | --- | --- | --- | --- | --- | --- |
| 1 | Olaniyan et al. (2018) | Yes | Yes | Yes | Yes | Yes | Unclear | No | Yes | Unclear | Yes | 7 (63.6) | Included |

**Cross-sectional studies**

| **No.** | **Study** | **1. Were the criteria for inclusion in the sample clearly defined?** | **2. Were the study subjects and the setting described in detail?** | **3. Was the exposure measured in a valid and reliable way?** | **4. Were objective, standard criteria used for measurement of the condition?** | **5. Were confounding factors identified?** | **6. Were strategies to deal with confounding factors stated?** | **7. Were the outcomes measured in a valid and reliable way?** | **8. Was appropriate statistical analysis used?** | **Score (percentile)** | **Overall appraisal:** |
| --- | --- | --- | --- | --- | --- | --- | --- | --- | --- | --- | --- |
| 1 | Amaechi et al. (2020) | Yes | Yes | Yes | Yes | No | Not applicable | Yes | No | 5 (62.5) | Included |
| 2 | Bisanzio et al. (2014) | Yes | Yes | Yes | Yes | No | Not applicable | Yes | Yes | 6 (75) | Included |
| 3 | Che et al. (2015) | Yes | Yes | Yes | Yes | No | Not applicable | Yes | Yes | 6 (75) | Included |
| 4 | Coulibaly et al. (2021) | No | Yes | Yes | Yes | No | Not applicable | Yes | Yes | 5 (62.5) | Included |
| 5 | Drame et al. (2016) | Yes | Yes | Yes | Yes | No | Not applicable | Yes | Yes | 6 (75) | Included |
| 6 | Ehounoud et al. (2021) | Yes | Yes | Yes | Yes | No | Not applicable | Yes | Yes | 6 (75) | Included |
| 7 | Ghosh et al. (1995) | Unclear | Yes | Yes | Yes | No | Not applicable | Yes | No | 4 (50) | Included |
| 8 | M’bondoukwé et al. (2022) | Yes | Yes | Yes | Yes | Yes | Yes | Yes | Yes | 8 (100) | Included |
| 9 | Mboera et al. (2011) | Unclear | Yes | Yes | Yes | No | Not applicable | Yes | Yes | 5 (62.5) | Included |
| 10 | Muturi et al. (2006) | Yes | Yes | Yes | Yes | No | Not applicable | Yes | Yes | 6 (75) | Included |
| 11 | Nielsen et al. (2007) | Yes | Yes | Yes | Yes | Yes | No | Yes | Yes | 7 (87.5) | Included |
| 12 | Prasad et al. (1990) | Yes | Yes | Yes | Yes | No | Not applicable | Yes | Yes | 6 (75) | Included |
| 13 | Ravindran et al. (1998) | No | Yes | Yes | Yes | No | Not applicable | Yes | No | 4 (50) | Included |
| 14 | Stensgaard et al. (2011) | Yes | Yes | Yes | Yes | Yes | Yes | Yes | Yes | 8 (100) | Included |
| 15 | Yoboue et al. (2022) | Yes | Yes | Yes | Yes | No | Not applicable | Yes | Yes | 6 (75) | Included |

**Prospective and retrospective observational studies**

| **No.** | **Study** | **1. Was the sample frame appropriate to address the target population?** | **2. Were study participants sampled in an appropriate way?** | **3. Was the sample size adequate?** | **4. Were the study subjects and the setting described in detail?** | **5. Was the data analysis conducted with sufficient coverage of the identified sample?** | **6. Were valid methods used for the identification of the condition?** | **7. Was the condition measured in a standard, reliable way for all participants?** | **8. Was there appropriate statistical analysis?** | **9. Was the response rate adequate, and if not, was the low response rate managed appropriately?** | **Score (percentile)** | **Overall appraisal:** |
| --- | --- | --- | --- | --- | --- | --- | --- | --- | --- | --- | --- | --- |
| 1 | Acharya et al. (2020) | Yes | Yes | Yes | Yes | Yes | Yes | Yes | No | Yes | 8 (88.9) | Included |
| 2 | Boumbanda Koyo et al. (2020) | Yes | Yes | Yes | Yes | Yes | Yes | Yes | Yes | Yes | 9 (100) | Included |
| 3 | Chadee et al. (2003) | Yes | Yes | Yes | Yes | Yes | Yes | Yes | No | Yes | 8 (88.9) | Included |
| 4 | Moutongo Mouandza et al. (2020) | Yes | Yes | Yes | Yes | Yes | Yes | Yes | Yes | Yes | 9 (100) | Included |
| 5 | Pousibet-Puerto et al. (2021) | Yes | Yes | Yes | Yes | Yes | Yes | Yes | Yes | Yes | 9 (100) | Included |
| 6 | Treeprasertsuk et al. (1998) | Yes | Yes | Yes | Yes | Yes | Yes | Yes | No | Yes | 8 (88.9) | Included |
